# Is training in basic echocardiography feasible in junior doctors?

**DOI:** 10.1186/2036-7902-4-S1-A13

**Published:** 2012-12-18

**Authors:** Julina Md Noor, F Mohd Salleh

**Affiliations:** 1 Faculty of Medicine, Universiti Teknologi MARA, Malaysia; 2 Emergency & Trauma Department, Sungai Buloh Hospital, Malaysia

## Background

In bedside ultrasound teaching, it has been generally accepted that Focused Assessment with Sonography in Trauma (FAST) is well received by medical students and junior doctors. However there is no evidence to date on the training of bedside basic echocardiography among junior doctors. As echocardiography is a quick and non-invasive diagnostic tool in the emergency department, the inclusion of this into junior doctors training should be a logical step.

## Objective

This study aims to demonstrate the feasibility of training basic echocardiography among junior doctors.

## Patients and methods

Junior doctors with no previous exposure or formal training in basic echocardiography were given an initial 5-hour theoretical and practical basic echocardiography training. They were given a pre and post assessment in the form of an objective structured clinical examination (OSCE). Following this, the doctors were required to complete 10 supervised echocardiography by emergency physicians trained in focused echocardiography. After 3 weeks, a theory and practical assessment was held by an independent observer. Participants also filled a feedback form on the perception of the training.

## Results

Results demonstrated that the junior doctors were able to perform satisfactorily and the level of confidence in performing basic echocardiography was higher after the training. They also felt that this training should be instituted for junior doctors and intended to seek further echocardiography training.

## Conclusion

Training basic echocardiography among junior doctors is feasible and increases performer confidence.
